# [⁶⁸Ga]Ga-FAPI-46 PET/CT imaging in neuromelioidosis: a case series demonstrating potential utility in diagnosis and response assessment

**DOI:** 10.1186/s41824-026-00284-w

**Published:** 2026-04-10

**Authors:** C. M. Mayur, Keerthana Kiran Kandula, M. A. Ashique Rahman, Suprava Naik, Bijayini Behera, Girish Kumar Parida, Sanjeev Kumar Bhoi, P. Sai Sradha Patro, Kanhaiyalal Agrawal

**Affiliations:** 1 Department of Neurology, All India Institute of Medical Sciences, Bhubaneswar, Odisha 751019 India; 2 Department of Nuclear Medicine, All India Institute of Medical Sciences, Bhubaneswar, Odisha 751019 India; 3 Department of Radiodiagnosis, All India Institute of Medical Sciences, Bhubaneswar, Odisha 751019 India; 4 Departmentof Microbiology, All India Institute of Medical Sciences, Bhubaneswar, Odisha 751019 India

**Keywords:** Burkholderia pseudo-mallei, Brain, Fibroblast activation protein, FDG, PET/CT

## Abstract

Neuromelioidosis is a rare but severe manifestation of melioidosis with central nervous system (CNS) involvement, caused by gram negative saprophytic bacteria named Burkholderia pseudo-mallei and is associated with high morbidity and mortality. Early and accurate detection of active disease is crucial for appropriate management. We report a series of three cases of neuromelioidosis evaluated using Gallium-68–labelled fibroblast activation protein inhibitor ([⁶⁸Ga]Ga-FAPI) positron emission tomography/computed tomography (PET/CT). [⁶⁸Ga]Ga-FAPI, a novel molecular imaging tracer targeting activated fibroblasts, has demonstrated utility in imaging infection and inflammation in addition to malignancies. Unlike [¹⁸F]FDG (F-18 Fluorodeoxyglucose), [⁶⁸Ga]Ga-FAPI shows negligible physiological uptake in normal brain parenchyma, allowing superior lesion conspicuity. In the three cases reported, [⁶⁸Ga]Ga-FAPI PET/CT clearly demonstrated active CNS inflammatory lesions better than [¹⁸F]FDG PET-CT, facilitating accurate disease assessment and aiding clinical decision-making. The findings on [⁶⁸Ga]Ga-FAPI PET/CT correlated with the MRI findings and further it was found to be better than MRI for response evaluation in one case. This highlights the potential role of [⁶⁸Ga]Ga-FAPI PET/CT as a promising functional imaging modality in the evaluation of neuromelioidosis and other CNS infections.

## Introduction

Melioidosis is a potentially fatal infectious disease caused by the saprophytic Gram-negative bacillus *Burkholderia pseudomallei*, commonly found in contaminated soil and water in tropical and subtropical regions, particularly Southeast Asia and Northern Australia (Gassiep et al. 2020). Several host factors such as diabetes mellitus, alcoholism, chronic liver or renal disease, and thalassemia, significantly increase the risk of acquiring melioidosis (Selvam et al. 2022). Infection typically occurs through inhalation, ingestion, or inoculation via abraded skin (Bzdyl et al. 2022). The disease exhibits a wide clinical spectrum, ranging from acute bacteraemia to disseminated abscesses, pneumonia, osteomyelitis, septicaemia, and, rarely, central nervous system (CNS) involvement (Hui et al. 2022). Neurological melioidosis (neuromelioidosis) occurs in ≤ 5% of patients and is associated with substantially higher morbidity and mortality, particularly when diagnosis or treatment is delayed (Currie et al. 2000).

Diagnostic confirmation relies on culture or cerebrospinal fluid (CSF) real-time polymerase chain reaction (RT-PCR), although these tests may be negative in early or partially treated infection (Currie et al. 2000). Imaging therefore plays a crucial role. Magnetic Resonance Imaging (MRI) findings of neuromelioidosis—such as ring-enhancing microabscesses, white-matter tract involvement (“tunnel sign”), meningeal enhancement, and extradural or skull lesions are characteristic but not pathognomonic (Naik et al. 2023; Kulkarni et al. 2020; Goldstein et al. 1997; Erol Fenercioğlu et al. 2023; Wang et al. 2023; Liu et al. 2024; Hsu et al. 2016; Agarwal et al. 2023). Anatomical imaging like MRI, while essential, may not reliably differentiate active infection from residual structural changes post-therapy.

Hybrid molecular imaging with F-18 Fluorodeoxyglucose Positron Emission Tomography/ Computed Tomography ([¹⁸F]FDG PET/CT) can assist in detecting systemic infectious foci (Kulkarni et al. 2020). It is the most common PET tracer for infection/inflammation imaging, due to increased glucose turnover in these conditions. However, in case of CNS infections, its diagnostic value is limited due to high physiological tracer uptake by the normal brain parenchyma.

Fibroblast Activating Protein Inhibitor (FAPI) is a type II transmembrane serine protease, which is known to be highly expressed by activated fibroblasts (Goldstein et al. 1997). Infectious and inflammatory processes recruit activated fibroblasts during tissue remodelling, leading to overexpression of FAP, which enables FAPI uptake. There are many case reports showing significant expression of FAP in infections and inflammatory conditions apart from malignancies (Erol Fenercioğlu et al. 2023; Wang et al. 2023). Gallium-68 Fibroblast Activation Protein Inhibitor [⁶⁸Ga]Ga-FAPI PET/CT is an emerging diagnostic modality that has shown promising results with more specific uptake compared to [¹⁸F]FDG PET/CT, particularly in CNS lesions due to no significant physiological uptake by brain (Liu et al. 2024).

Here, we present a series of three cases, with clinical diagnosis of neuromelioidosis, who underwent both [⁶⁸Ga]Ga-FAPI-46 and ^18^F-FDG PET/CT brain imaging, highlighting the characteristic uptake patterns of [⁶⁸Ga]Ga-FAPI-46 PET/CT, as compared with [¹⁸F]FDG PET/CT and MRI, thus describing its potential utility in diagnosis, disease mapping, and post treatment response assessment.

## Case presentations

### Case 1

A 17-year-old male student from costal region of India, developed high-grade and continuous fever associated with headache for 10 days. Gradually, he developed right lower motor neuron (LMN) facial palsy and dysphagia to both solids and liquids. Later, he developed left hemiparesis and double vision. On admission, he was conscious and oriented with stable vitals but had dyspnoea and ataxic breathing requiring intubation. Other systemic examination findings were normal. His past medical history included a road traffic accident 3 months prior, resulting in a lacerated injury over the left parietal region.

Laboratory investigations revealed normal routine blood parameters. Cerebrospinal fluid (CSF) analysis showed 18 cells (lymphocyte predominant), protein of 58 mg/dL (normal 15–45 mg/dl), and glucose of 59 mg/dL (normal 50–80 mg/dl), suggestive of infective etiology. Further CSF analysis ruled out tubercular, common bacterial and viral infections.

**Fig. 1:**
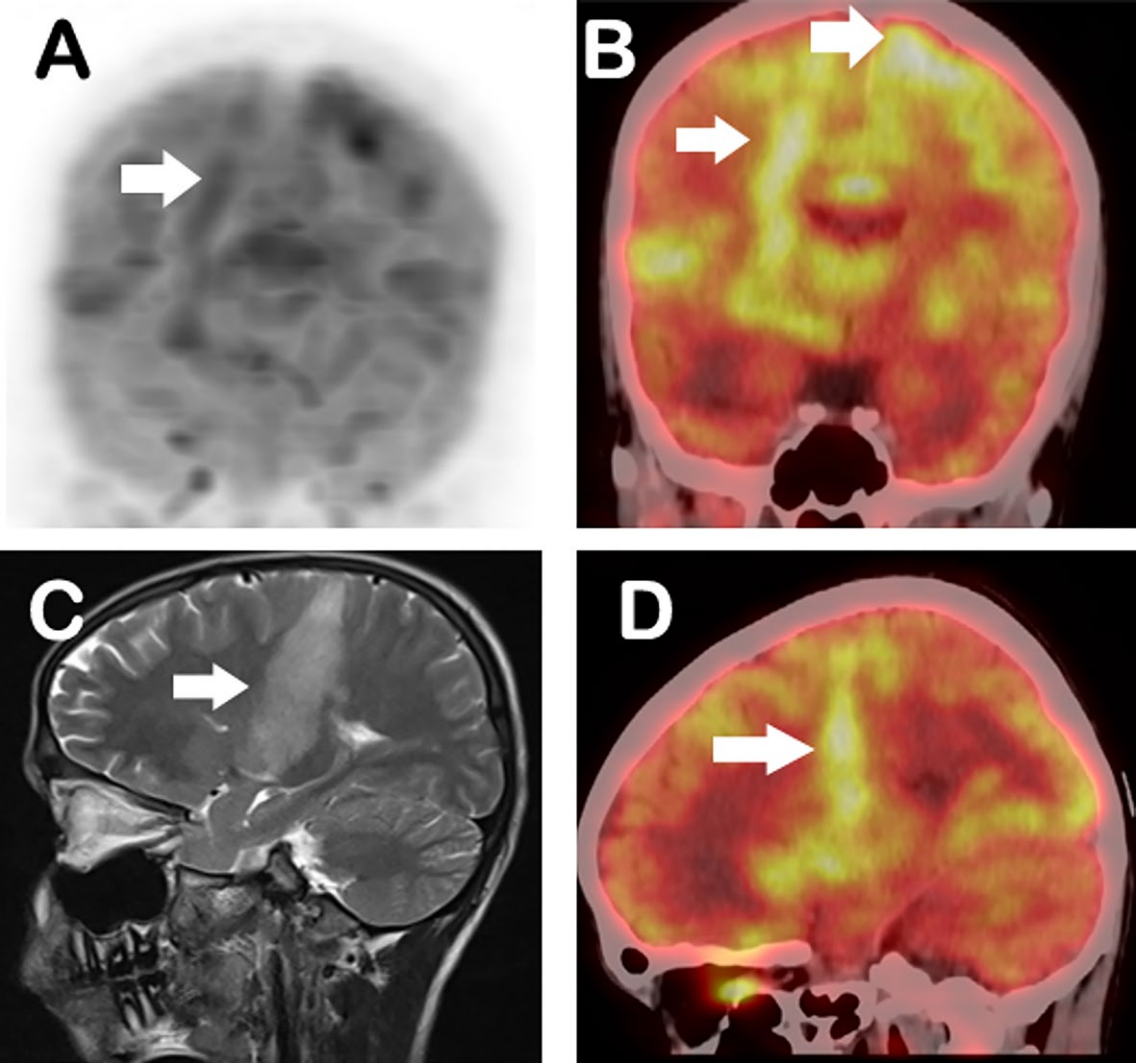
17-year-old boy with brain SOL (**A**) ^18^F-FDG PET/CT MIP (Maximum intensity projection), (**B, D**) sagittal and coronal fused PET/CT images show minimally FDG avid (SUVmax 7.1) ill-defined linear hyperdense areas involving right frontal lobe, right basal ganglia, and right thalamus extending till right cerebral peduncle of midbrain, with perilesional edema (arrows **A, B, D**). Another Focal FDG avid subtle hyper density is noted in the left high frontal cortex, SUVmax 9.1 (arrows in **B**). (**C**) MRI sagittal view demonstrated T2 hyperintensity along the corticospinal tract from the cortex to the subcortex, tracking along the white matter tract to the midbrain, a finding termed as “Tunnel Sign” (arrow in **C**)

Further, Regional [¹⁸F]FDG PET/CT of brain was performed 60 min after intravenous injection of 3.7 MBq/kg (0.1 mCi/kg) of the radiotracer, following 6 h of fasting and confirming blood glucose < 160 mg/dL, on Discovery MIDR PET/CT scanner (GE Medical Systems, Milwaukee, Wisconsin, USA). [¹⁸F]FDG PET/CT of brain showed an ill-defined FDG avid (SUVmax 7.1) linear hyperdense lesion involving the right frontal lobe, basal ganglia, and thalamus extending along white matter tracts till the right cerebral peduncle of the midbrain and associated with perilesional edema. Another focal FDG uptake with ill-defined subtle hyper density was noted in the left high frontal cortex (SUVmax 9.1). These were more in favour of infective etiology. Even though FDG uptake of the lesions were slightly above the physiological uptake of brain (SUVmax- 5.3) but the uptake appeared to be smeared with the high background**/** physiological brain activity. (Fig. 1)

Contrast-enhanced magnetic resonance imaging (MRI) of the brain and spinal cord revealed T2/FLAIR hyperintensity in the right perirolandic region, right centrum semiovale, internal capsule, and right thalamus, with an abscess in the right midbrain. Sagittal view of the MRI demonstrated T2 hyperintensity along the corticospinal tract from the cortex to the subcortex, tracking along the white matter tract to the midbrain, a finding termed as “Tunnel Sign”, a specific sign for Neuromelioidosis (Fig. 1C).

**Fig. 2:**
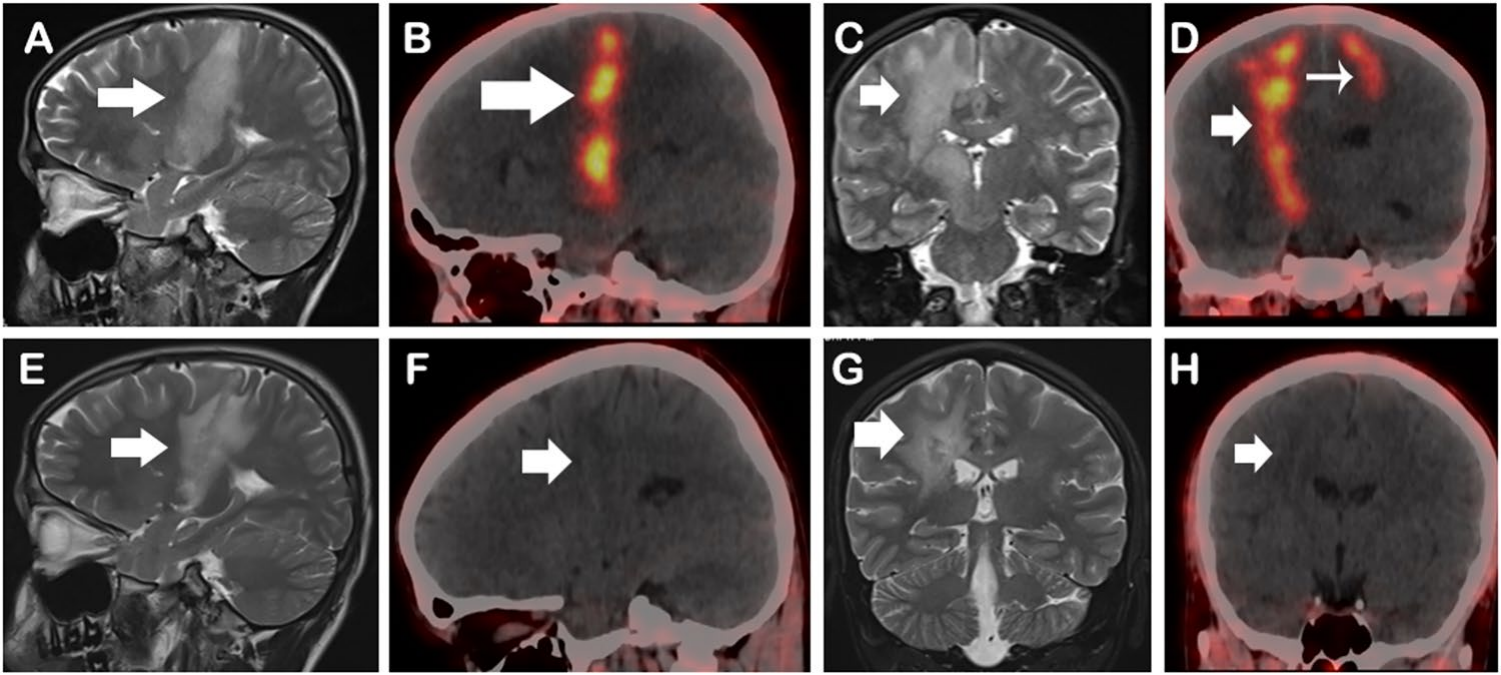
Corresponding MRI and ^68^Ga- FAPI PET/CT images in the same patient mentioned in Fig. 1, before (**A-D**) and after treatment (**E-H**). MRI T2/FLAIR sagittal and coronal images, demonstrated T2 hyperintensity along the corticospinal tract from the cortex to the subcortex, tracking along the white matter tract to the midbrain, a finding termed as “Tunnel Sign” (arrows in **A, C**). ^68^Ga FAPI PET/CT sagittal and coronal images show, tracer-avid (SUVmax 5.6) linear hyperdense areas involving the right frontal lobe, extending to the right basal ganglia and thalamus with significant perilesional edema (arrows in **B, D**). Another focal tracer uptake with a subtle hyperdense lesion in the left high frontal cortex, SUVmax 3.1, which is better appreciated on FAPI PET-CT (thin arrow in **D**). Follow-up MRI images post treatment completion show residual anatomical changes (arrows in **E, G**). However, there is complete resolution of activity on corresponding FAPI PET-CT images (arrows **F, H**). Patient improved significantly clinically post treatment

[⁶⁸Ga]Ga-FAPI-46 PET/CT was performed within a 5-day span, after injecting 74 Mbq (2 mCi) of ^68^Ga-FAPI-46 intravenously and without any special preparation. PET/CT scan of brain was acquired after 45 min of tracer injection in the same scanner. [⁶⁸Ga]Ga-FAPI-46 PET/CT images showed tracer avidity in the lesions documented on [¹⁸F]FDG PET/CT (with SUVmax 5.6). The lesion contrast was better on [⁶⁸Ga]Ga-FAPI-46 PET/CT than FDG due to negligible physiological background uptake in the brain (Fig. 2, A-D).

Based on the comprehensive clinical, CSF, and radiological findings, a diagnosis of neuromelioidosis was established. The patient was treated accordingly and improved symptomatically. Repeat [⁶⁸Ga]Ga-FAPI-46 PET/CT scan post treatment showed near complete resolution of the tracer avidity, suggestive of good treatment response, however MRI still showed residual structural disease (Fig. 2, E-H).

### Case 2

A 53-year-old woman, from rural part of India, presented with chief complaints of headache for 2 months, recurrent high-grade fever, associated with chills and rigors fever for 15 days and abnormal involuntary movements in both upper limbs for 5 days. Her headache was severe, subacute in onset, episodic and holocranial in nature. The headache was associated with neck pain that limited head movement, not associated with nausea, vomiting, blurring of vision, or double vision. On the 10th day of fever, she experienced one episode of tonic-clonic seizure lasting for 30–40 s, associated with confusion and body pain.

On examination, the patient was conscious and oriented with stable vitals. Other systemic examinations were normal with no cranial nerve palsies, normal muscle tone and reflexes.

Routine investigations revealed anaemia with leucocytosis. CSF analysis revealed 7 cells (predominantly lymphocytes), protein of 92 mg/dL (normal 15–45 mg/dl), and glucose of 83.6 mg/dL (normal 50–80 mg/dl) suggestive of infective etiology and ruled out tuberculosis/malignancy. Blood and mediastinal lymph node transbronchial needle aspiration (TBNA) culture were positive for *Burkholderia pseudomallei*.

**Fig. 3:**
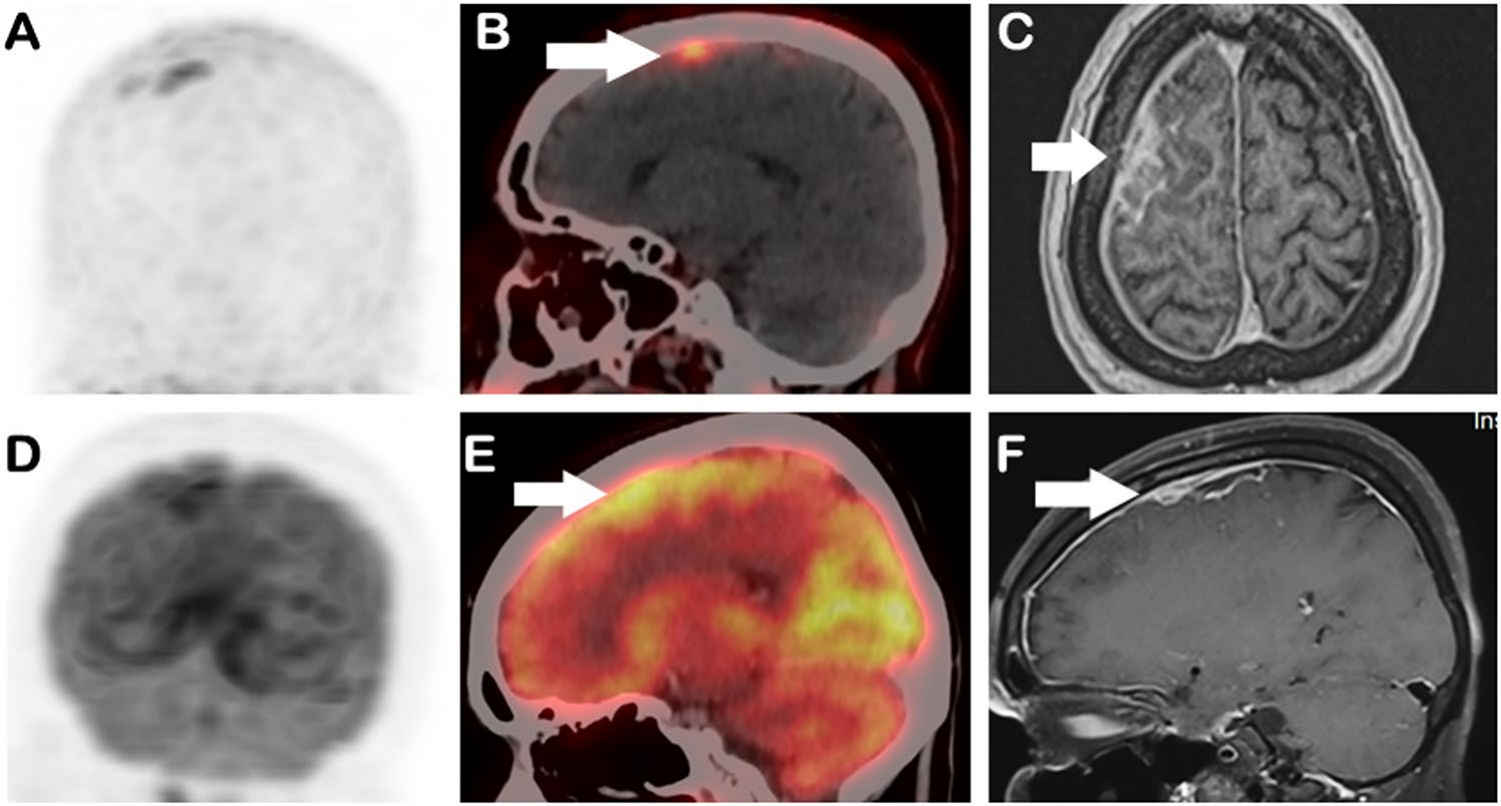
53-year-old female with Neuromelioidosis. MIP and sagittal fused ^68^Ga- FAPI PET/CT images shows focal tracer-avid meningeal enhancement in the right cerebral hemisphere along the right frontoparietal lobes with SUVmax 6.5 (**A, B**). Corresponding ^18^F-FDG PET/CT images shows mild FDG avid ill-defined enhancement in the same region with SUVmax 13.1, but not clearly distinct from the background uptake (**D, E**). MRI axial and sagittal post-contrast T1-weighted images shows diffuse pachymeningeal enhancement along the right cerebral hemisphere frontoparietal region confirming the disease involvement (arrows in **C, F**)

Brain [¹⁸F]FDG PET/CT was performed after an overnight fast of 6 h and confirmation of blood glucose < 160 mg/dL. A dose of 3.7 MBq/kg (0.1 mCi/kg) of [¹⁸F]FDG was administered intravenously, and imaging was acquired 60 min post-injection on a Discovery MIDR PET/CT scanner (GE Medical Systems, Milwaukee, USA). Images showed tracer avid (SUVmax 13.1) ill-defined enhancement along the right fronto-parietal region of brain, but it was not clearly distinct from the background brain uptake (SUVmax 11.3). Within 7 days of [¹⁸F]FDG PET/CT, [⁶⁸Ga]Ga-FAPI-46 PET/CT of brain was acquired 45 min after injection of 74 Mbq (2 mCi) of [⁶⁸Ga]Ga-FAPI intravenously. Images revealed focal tracer-avid meningeal enhancement in the right cerebral hemisphere along the right frontoparietal lobes (SUVmax 6.5), suggestive of infective etiology. The uptake in the lesion was more distinct than FDG, as there was negligible background tracer uptake by brain (Fig. 3).

MRI Brain demonstrated diffuse pachymeningeal enhancement along the right cerebral hemisphere and basal pontine region, with T2/FLAIR hyperintensity along the right frontal subcortical white matter, without diffusion restriction or post-contrast enhancement. (Fig. 3, e-f). Findings consistent with neuromelioidosis in the appropriate clinical setting. The patient was subsequently treated and discharged in stable condition.

### Case 3

38 years-old-male, farmer from India, presented with high grade continuous fever, associated with chills, left sided weakness, altered sensorium and headache for last 10 days. Headache was holocranial, initially mild dull aching and over days it progressed, severe enough to hamper his daily activities. It was associated with episodes of projectile vomiting and irritability, photophobia, drowsiness, weakness and delayed response. Patient was treated as a case of subacute meningoencephalitis initially. His MDCT head, showed well defined rim enhancing hypodense lesions with surrounding vasogenic edema and poor perilesional incomplete contrast enhancement in right anterior centrum semiovale and right high juxta cortical frontal lobe. A possibility of infective granuloma was raised.

The CSF analysis study was compatible with infection (Glucose-20.8 mg/dl, Protien-80.60 mg/dl, total counts- 900/cumm with 70% polymorphs). However, there was no organism grown in aerobic CSF culture.

**Fig. 4:**
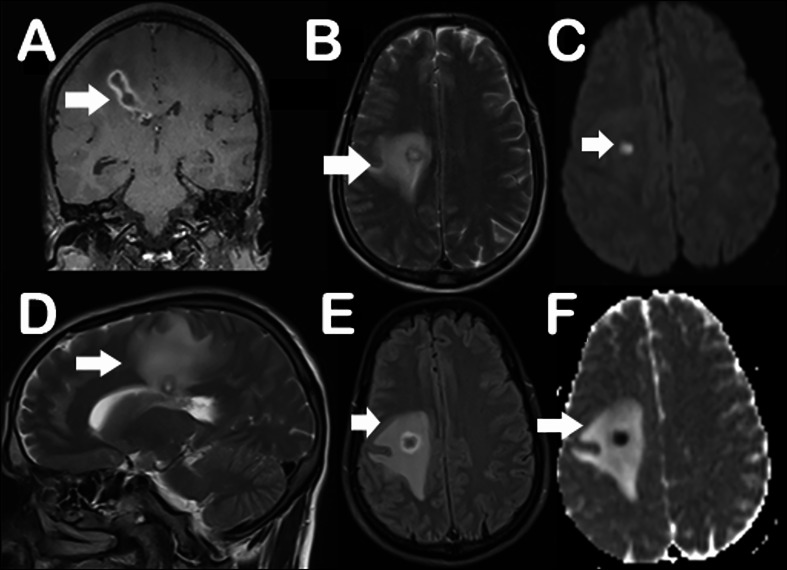
MRI images of 38-year-old male with Neuromelioidosis. Coronal MPRAGE sequence of MRI brain shows an irregular lesion with peripheral hyperintense signal in the right frontal cortex (arrow in **A**). T2 axial, sagittal and FLAIR sequences showing hyperintense peripherally enhancing lesion extending along the white matter to the body of corpus callosum (arrows in **B, D-E**). ADC and DWE axial images showing central diffusion restriction in the corresponding lesion (arrows in **C, F**)

Subsequently he underwent MRI brain which revealed multiple conglomerated T2/FLAIR hyperintense peripherally enhancing lesions with central diffusion restriction and blooming in the wall in the right posterior frontal lobe. The lesion was extending along the fibres of corona radiata into the body of corpus callosum and into the right lateral ventricle, a finding termed as “Tunnel Sign”. Similar lesions were also seen in the right choroid plexus. The findings were consistent with brain abscesses and raised a high possibility of neuromelioidosis (Fig. 4).

**Fig. 5:**
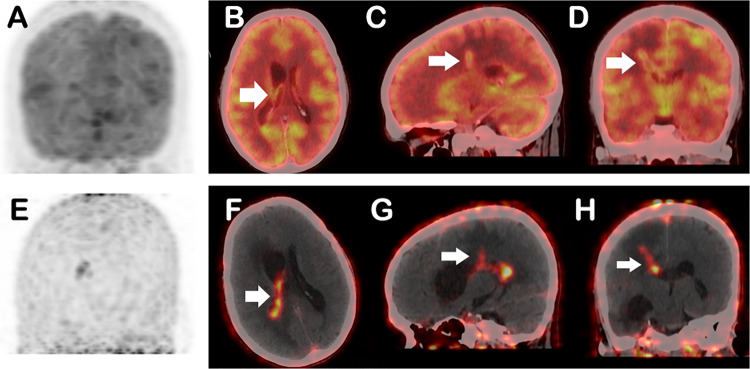
Corresponding ^18^F-FDG PET/CT (**A-D**) and ^68^Ga- FAPI PET/CT (**E-H**) images in the same patient mentioned in Fig. 4. Maximum intensity projection FDG PET image of the brain showing no definite abnormal uptake (**A**). Axial, sagittal and coronal ^18^F-FDG PET/CT images show ill-defined peripherally FDG avid rim enhancing lesions in the right frontal cortex with surrounding vasogenic oedema formic tract and crossing adjacent lateral ventricle with involvement of choroid plexus (arrows in B-D). Maximum intensity projection ^68^Ga- FAPI PET image of the brain shows heterogenous tracer uptake in the right cerebral cortex. Axial, sagittal and coronal ^68^Ga- FAPI PET/CT images showing FAP expressing lesions in the right frontal cortex with surrounding vasogenic oedema forming tract and crossing adjacent lateral ventricle and involving choroid plexus (arrows in **F-H**)

About 3.7 MBq/kg (0.1 mCi/kg) of [¹⁸F]FDG was injected intravenously, after a fasting period of about 6 h and brain PET/CT images were acquired 60 min post-injection on Discovery MIDR PET/CT scanner (GE Medical Systems, Milwaukee, Wisconsin, USA). Images revealed peripherally tracer avid rim enhancing lesion (with SUVmax- 4.8, similar to background SUVmax- 4.4) involving the right frontal cortex forming a tract till right lateral ventricle, extending along choroid plexus with associated vasogenic edema and dilatation of right ventricle. Few other sub centimetric nodular enhancing lesions with FDG uptake similar to background were also noted in right frontal and parietal cortices. The findings were compatible with infective etiology (Fig. 5).

Further, [⁶⁸Ga]Ga-FAPI PET/CT of brain was acquired 45 min after injection of 74 Mbq (2 mCi) of [⁶⁸Ga]Ga-FAPI-46 intravenously. The appearance of lesions was similar to that on [¹⁸F]FDG PET/CT showing tracer avidity (SUVmax- 3.3), but [⁶⁸Ga]Ga-FAPI PET/CT showed better lesion to background contrast (Fig. 5).

The real time PCR (RT-PCR) assay was tested positive for T3SS1 gene and a microbiological confirmation of Burkholderia pseudomallei infection was made. After a final diagnosis of neuromelioidosis was made, patient was treated accordingly and improved symptomatically.

## Discussion

Neurological involvement occurs in fewer than 5% of melioidosis cases, yet it carries a high risk of mortality, exceeding 20% when untreated (Currie et al. 2000). Although CSF culture remains the diagnostic gold standard, it may be negative due to delayed or absent seroconversion, making imaging a key component of evaluation. Anatomical imaging, particularly MRI, plays a crucial role in diagnosing neuromelioidosis. Reported findings include ring-enhancing lesions (corresponding to micro-abscess) in the brain parenchyma, or hyperintense lesions tracking along the white matter tracts called as tunnel sign; in addition, meningeal enhancement, extradural and scalp abscesses, and skull osteomyelitis may be present (Naik et al. 2023; Kulkarni et al. 2020; Goldstein et al. 1997; Erol Fenercioğlu et al. 2023; Wang et al. 2023; Liu et al. 2024; Hsu et al. 2016; Agarwal et al. 2023). While MRI is highly sensitive for detecting abscesses, meningitis, and tract involvement, it lacks specificity and often cannot differentiate active infection from residual structural abnormalities during post treatment follow-up.

Molecular imaging shows early changes, particularly helpful in diagnosis and response assessment of neuromelioidosis. [¹⁸F]FDG PET/CT is widely used in infectious and inflammatory imaging; however, its utility in CNS infections is limited by the inherently high physiological FDG uptake in the cerebral cortex, which obscures subtle or small lesions and reduces lesion-to-background contrast. In this case series also, although lesions demonstrated mildly increased FDG uptake, the target-to-background ratio remained low, limiting diagnostic clarity.

A tracer with negligible physiological brain uptake could therefore provide substantial diagnostic benefit. Prior studies have highlighted the utility of [⁶⁸Ga]Ga-FAPI-46 PET/CT in infectious and inflammatory conditions due to its affinity for activated fibroblasts (Erol Fenercioğlu et al. 2023; Wang et al. 2023). Importantly, FAPI demonstrates minimal or no physiological uptake in normal brain parenchyma, enabling clear visualization of intracranial lesions. In all three patients in this series, [⁶⁸Ga]Ga-FAPI-46 PET/CT showed distinct tracer uptake with excellent lesion-to-background contrast, outperforming FDG PET/CT in delineating disease extent.

Beyond diagnosis, [⁶⁸Ga]Ga-FAPI-46 PET/CT proved valuable for treatment response assessment. Follow-up FAPI imaging showed complete metabolic resolution in one patient despite residual structural changes on MRI, suggesting its potential as a sensitive marker of active inflammation.

Compared with [¹⁸F]FDG which is limited by intense physiological cortical uptake [⁶⁸Ga]Ga-FAPI-46 provides a substantially higher lesion-to-background ratio, facilitating clearer detection of parenchymal, meningeal, and tract-based lesions, even when small or infiltrative.

Neuromelioidosis is rare, and FAPI imaging in this context remains exploratory; hence, only three patients who underwent dual-tracer PET/CT within routine clinical care were included. The primary limitations of this work are the small sample size and lack of histopathological confirmation for all lesions. These findings, while promising, require validation in larger prospective studies.

## Conclusion

[⁶⁸Ga]Ga-FAPI PET/CT appears a more promising tool in the initial assessment and also for post treatment response assessment in neuromelioidosis than the present standard diagnostic imaging modalities in this case series.

## Data Availability

The dataset generated during and/or analysed during the current study are available from the corresponding author on reasonable request.

## Declarations

### Submission declaration

This is authors original/own work. Full paper or part of it has not been submitted elsewhere for publication.

## Abbreviations

PET/CT: Positron Emission Tomography/ Computed Tomography
[⁶⁸Ga]Ga: Gallium-68
[¹⁸F]FDG: Fluorine-18 Fluorodeoxyglucose
CNS: Central Nervous System
FAPI: Fibroblast Activating Protein Inhibitor
LMN: Lower motor neuron
CSF: Cerebrospinal fluid
NCCT: Non-contrast computed tomography
MRI: Magnetic Resonance Imaging
